# Patients’ and health professionals’ understanding of and preferences for graphical presentation styles for individual-level EORTC QLQ-C30 scores

**DOI:** 10.1007/s11136-015-1107-3

**Published:** 2015-09-09

**Authors:** W. Kuijpers, J. M. Giesinger, A. Zabernigg, T. Young, E. Friend, I. M. Tomaszewska, N. K. Aaronson, B. Holzner

**Affiliations:** Division of Psychosocial Research and Epidemiology, The Netherlands Cancer Institute, Amsterdam, The Netherlands; Department of Psychiatry and Psychotherapy, Medical University of Innsbruck, Innsbruck, Austria; Department of Internal Medicine, Kufstein County Hospital, Kufstein, Austria; Lynda Jackson Macmillan Centre, Mount Vernon Cancer Center, Northwood Middlesex, UK; Hepatobiliary Research, Basingstoke and North Hampshire Hospital, Basingstoke, Hampshire UK; Department of Medical Education, Jagiellonian University Medical College, Kraków, Poland

**Keywords:** Patient-reported outcomes, Graphical presentation, Cancer patients, Health professionals

## Abstract

**Purpose:**

To investigate patients’ and health professionals’ understanding of and preferences for different graphical presentation styles for individual-level EORTC QLQ-C30 scores.

**Methods:**

We recruited cancer patients (any treatment and diagnosis) in four European countries and health professionals in the Netherlands. Using a questionnaire, we assessed objective and self-rated understanding of QLQ-C30 scores and preferences for five presentation styles (bar and line charts, with or without color coding, and a heat map).

**Results:**

In total, 548 patients and 227 health professionals participated. Eighty-three percent of patients and 85 % of professionals self-rated the graphs as very or quite easy to understand; this did not differ between graphical presentation styles. The mean percentage of correct answers to questions objectively assessing understanding was 59 % in patients, 78 % in medical specialists, and 74 % in other health professionals. Objective understanding did not differ between graphical formats in patients. For non-colored charts, 49.8 % of patients did not have a preference. Colored bar charts (39 %) were preferred over heat maps (20 %) and colored line charts (12 %). Medical specialists preferred heat maps (46 %) followed by non-colored bar charts (19 %), whereas these charts were equally valued by other health professionals (both 32 %).

**Conclusion:**

The substantial discrepancy between participants’ high self-rated and relatively low objective understanding of graphical presentation of PRO results highlights the need to provide sufficient guidance when presenting such results. It may be appropriate to adapt the presentation of PRO results to individual preferences. This could be facilitated when PROs are administered and presented to patients and health professionals electronically.

## Introduction

Patient-reported outcomes (PROs) are frequently used as outcome measures in cancer clinical trials and in observational studies. More recently, they have also been introduced into daily clinical practice, where they provide clinicians and nurses with information about the symptom experience, functional health, and subjective well-being of patients that can be used during the clinical encounter. Although this feedback from PROs often leads to improved symptom detection [1–3], more discussion of problems [1–3], and higher levels of patient satisfaction [2], only a few studies have found a direct impact on quality of life (QoL) [4, 5].

Electronic data collection systems have been developed to facilitate the introduction of PROs in daily clinical practice. The major advantages of these electronic systems are that they facilitate efficient data collection and that PRO results are directly available [6]. Most recently, PRO data collection systems have been integrated into Web-based patient portals and can be integrated into the electronic medical record. The use of an electronic data collection system facilitates graphical presentation of the PRO results. Graphs are especially useful for the display of dynamic data, such as change over time [7].

To date, only limited information is available regarding how best to graphically summarize and display the results of PROs for both patients and health professionals. Several studies have investigated patients’ and health professionals’ understanding of graphically presented quality-of-life data at the group level, as obtained in clinical trials. These studies have shown that patients are most accurate in interpreting simple line graphs compared to simple bar charts or more complex graphs [8, 9] and that professionals prefer line graphs presenting change over time [10].

Individual PRO results are most likely to be presented as absolute scores at fixed time points. Although this allows for calculating and displaying change over time, the interpretation of an absolute score at a single time point is more challenging. The interpretation of absolute scores can be facilitated through the use of clinical thresholds that allow one to classify individual patients as a “case” [11]. The caseness thresholds may reflect a priori decision rules regarding symptom severity or may be related to external criteria or percentiles from general population or patient reference groups. Such thresholds can be integrated into graphical displays of PRO results using color-coding methods that indicate the severity or clinical importance of a symptom or problem [12–14].

Given the paucity of studies on the graphical presentation of individual-level PRO results, the aim of the current study was to investigate patients’ and health professionals’ understanding of and preferences for different graphical presentation styles for the EORTC QLQ-C30, a questionnaire frequently used to assess QoL in cancer patients [15]. In addition, we asked patients and health professionals their opinions about general aspects of PRO data collection and use in daily clinical practice.

## Methods

### Patient survey

#### Procedures

**Fig. 1:**
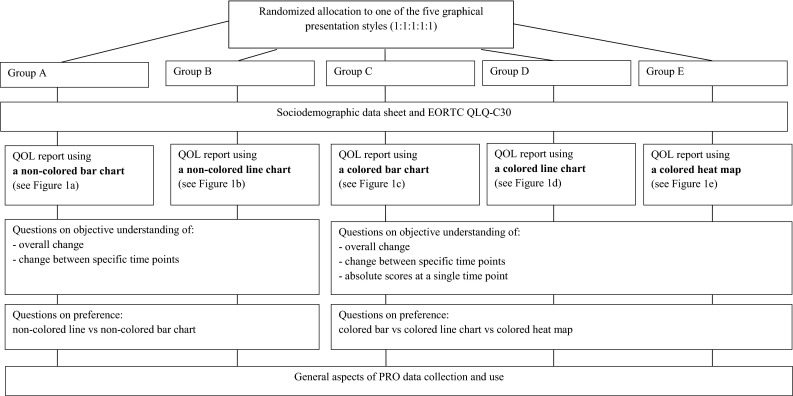
Flowchart of the patient questionnaire

A cross-sectional sample was recruited from the Netherlands Cancer Institute (the Netherlands), Mount Vernon Cancer Centre, Basingstoke & North Hampshire Hospital (both United Kingdom), Kufstein County Hospital (Austria), and the Jagiellonian University Medical College (Poland). We aimed to obtain a heterogeneous sample consisting of patients with any type of cancer who were receiving or had received treatment (chemotherapy, radiation therapy, surgery). Patients were approached by mail (followed by a reminder by mail) or at the outpatient clinic and were asked to complete a questionnaire. The institutional review board of each participating center approved the study following local standards, and patients provided written informed consent where required. We obtained clinical data (cancer site, cancer stage) from the (electronic) medical record and sociodemographic data (age, sex, marital status, education) via the questionnaire. Patients were randomly assigned to one of five versions of this questionnaire. The allocation of patients and the topic areas covered by the study questionnaire are summarized in Fig. 1.

#### EORTC QLQ-C30

Patients first completed the EORTC QLQ-C30 to become familiar with a quality-of-life instrument. The QLQ-C30 contains five functioning scales (physical, social, role, cognitive, and emotional), nine symptom scales (fatigue, nausea/vomiting, pain, dyspnea, sleeping disturbances, appetite loss, constipation, diarrhea, and financial impact), and a global QoL scale [15]. Response choices range from 1 (not at all) to 4 (very much), with the exception of the two items of the global QoL scale, where responses range from 1 (very poor) to 7 (excellent). All scale scores are linearly transformed to a 0–100 scale. For the functioning scales and the global QoL scale, a higher score represents a higher level of functioning or QoL. For the symptom scales, a higher score represents more symptom burden.

#### Graphical presentation styles

**Fig. 2:**
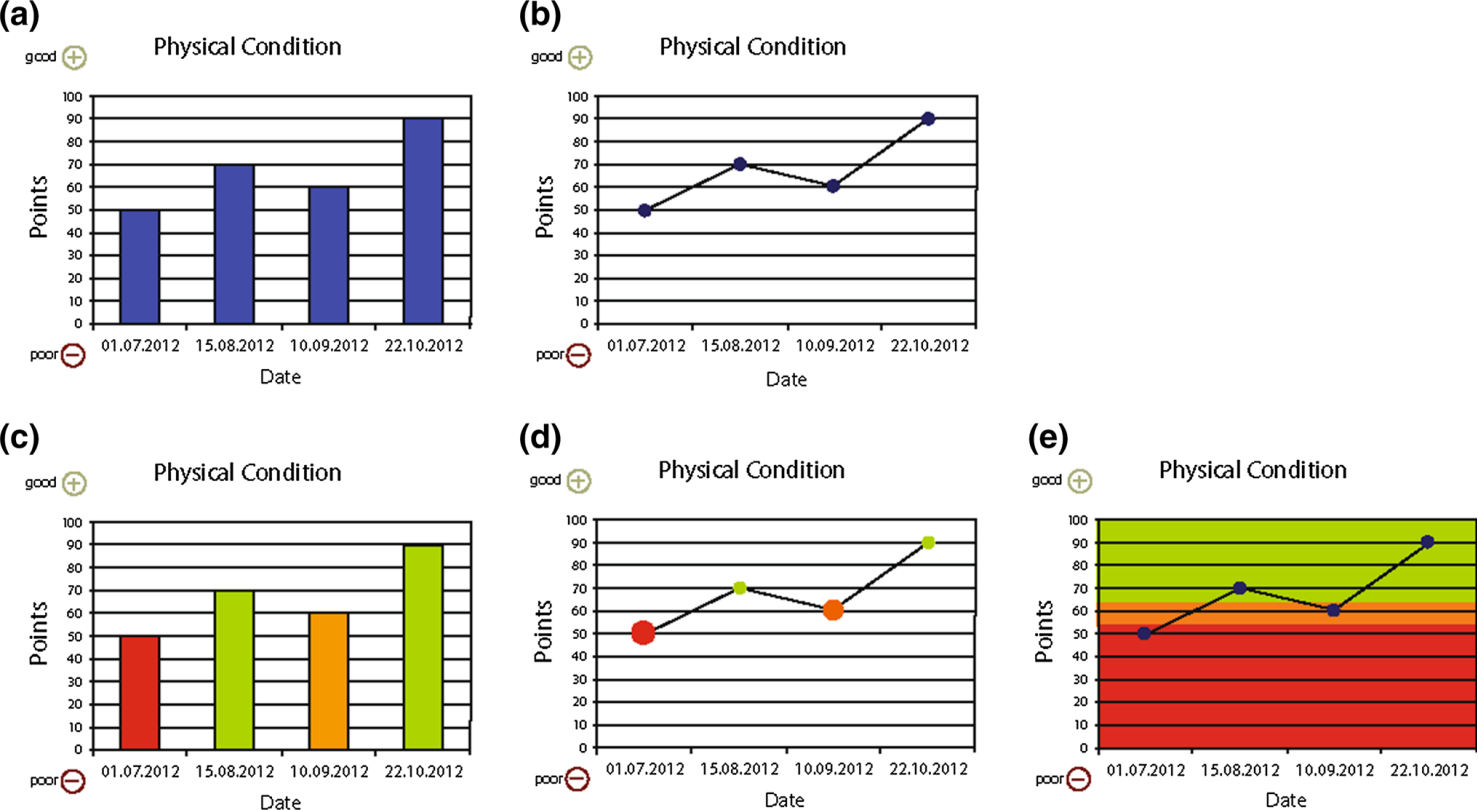
Five graphical presentation styles for physical functioning used in this study

Based on their randomization, patients received one of five frequently used graphical presentation styles: non-colored and colored bar charts and line charts, and a heat map (see Fig. 2a–e).

We included both non-colored and colored charts to assess preferences separately for charts using and not using color-coded reference values. The graphs contained hypothetical data from four assessment points for four QLQ-C30 scales: physical and emotional functioning, fatigue, and pain. For each graph, we assessed the *objective* understanding of absolute scores by asking patients to rate the extent of the problem (none/mild, moderate or severe). These questions on absolute scores were only presented to patients who received a survey with colored graphs. To assist patients in understanding the graphs, we added a description explaining the meaning of the colors (green—no/mild; orange—moderate; red—severe). *Objective understanding* of change scores was assessed with these questions: “Overall, over the 4 time points, how did health status change?” (overall change; continuous worsening, continuous improvement, fluctuation between improvement and worsening) and “How did the health status change from 10.09.2012 to 22.10.2012?” (specific change; worsened, no change, improved). We assessed *subjective* understanding with the following question: “How easy or difficult was it for you to understand the graphs?” (1–4 scale, ranging from very easy to very difficult).

Regarding preferences, we made a distinction between non-colored and colored charts. Patients had to choose from among the two non-colored charts or from among the three colored charts, depending on the questionnaire they received (i.e., they were not given all five charts, but just the two non-colored or the three colored ones). We also asked for their preference regarding three different directional indicators (indicating whether a score of 100 is good or bad): plus and minus signs (see Fig. 2a–e), green and red arrows or a green arrow pointing in the direction of better scores.

#### General aspects of PRO data collection and use

The last section of the questionnaire consisted of general questions on the use of PROs in clinical practice (e.g., “How often would you be willing to complete a questionnaire during treatment about how you are feeling?”).

### Health professional survey

#### Procedures

All health professionals from the Netherlands Cancer Institute involved in patient care were eligible to participate. They were invited via email to complete an online questionnaire using SurveyMonkey [16] about the use an interpretation of PROs. Reminders were sent after 3 weeks.

#### Sociodemographics and introduction

The survey began with questions about participant characteristics and previous experience with QoL data. The EORTC QLQ-C30 was displayed to familiarize the respondents with such questionnaires and to provide a context for the remainder of the survey.

#### Graphical presentation styles

We introduced the five graphs (both non-colored and colored; Fig. 2a–e) and asked the respondents to rank them in order of preference. Each respondent was then shown graphs with data for four assessment points for the QLQ-C30 physical functioning and fatigue scales in the format which (s)he had indicated was his/her first preference. The questions to assess professionals’ objective and subjective understanding of the graphically presented QLQ-C30 data were identical to those posed to patients. We also asked for their preference regarding different directional indicators and provided space for general comments.

#### General aspects of PRO data collection and use

Finally, we asked the health professionals whether they believe that PRO questionnaires are useful for obtaining information about the health status and well-being of their patients. If the answer was positive, we posed follow-up questions on the use of PROs in clinical practice (e.g., “How often would you like to receive PRO information about a patient during treatment?”; “Do you think it is useful to have access to PRO results via the electronic medical record?”). If the initial response regarding usefulness of PROs was negative, they were asked to explain their rating.

### Statistical analyses

We used descriptive statistics for sociodemographics, clinical data, and the general questions. For each question assessing understanding, we calculated the percentage of participants with a correct answer. In addition, we calculated the mean number of correct answers for questions on the understanding of absolute scores, for questions on change over all four time points, for questions on specific change and a total score of all correct answers. For patients, the maximum number of correct answers was 12 (4 domains × 3 questions) and for health professionals 6 (2 domains × 3 questions). We used Kruskal–Wallis tests to compare the number of correct answers to the questions assessing absolute scores, overall change, and specific change as a function of the graphical presentation styles. We used the Mann–Whitney *U* test to assess differences in understanding as a function of profession (medical specialists versus nurses and other health professionals combined). Using t-tests, we compared differences in understanding of functioning scales and symptom scales, in understanding of professionals with and without previous experience with PROs, in understanding of men versus women, and in understanding of younger versus older participants. One-way ANOVA was used to test whether objective understanding was influenced by educational level. We calculated the percentage of respondents who expressed a preference for each of the graphical presentation styles and used the Chi-square statistic to assess differences in preferences. We considered *p*-values below 0.05 to be statistically significant. All analyses were performed using the Statistical Package for the Social Sciences (SPSS) version 22.

## Results

### Patient survey

#### Sociodemographics

A total of 548 patients participated (the Netherlands *N* = 236, Austria *N* = 151, Poland *N* = 100, the UK *N* = 61). The mean age was 60.6 years (SD 12.3), and 54 % was female. The most common diagnoses were breast cancer (25.7 %), colorectal cancer (12.8 %), and lung cancer (11.7 %), and 61.2 % of patients had UICC stage III or IV disease. Approximately three-quarter of the patients were on active treatment at the time they completed the questionnaire. Further patient characteristics are shown in Table 1.

**Table 1:** Patient characteristics (*N* = 548)

Age	
Mean (SD)	60.6 (12.3)
Range	19–89
Sex	
Female	54.0 %
Male	46.0 %
Education	
Compulsory or less	31.2 %
Post compulsory	41.6 %
University or college	27.3 %
Employment status	
Full-time job	20.4 %
Part-time job	10.2 %
Homemaker	7.4 %
Retired	36.3 %
Unemployed	3.7 %
Student	0.2 %
Other	11.8 %
UICC stage	
I	9.9 %
II	28.9 %
III	24.4 %
IV	36.8 %
Cancer site (primary)	
Breast cancer	25.7 %
Colorectal cancer	12.8 %
Lung cancer	11.7 %
Head and neck cancer	8.0 %
Prostate cancer	7.4 %
Non-Hodgkin lymphoma	7.3 %
Stomach/esophageal cancer	6.6 %
Gynecologic cancer	6.0 %
Other	14.5 %
Treatment status	
On-treatment	74.3 %
Off-treatment	25.7 %

#### Graphical presentation styles

Randomized allocation of patients to the five graphical presentation styles resulted in 20.6 % of the patients receiving non-colored bar charts, 21.7 % receiving non-colored line charts, 19.7 % receiving colored bar charts, 18.6 % receiving colored line charts, and 19.3 % receiving heat maps (see Fig. 2a–e).

Table 2 shows patients’ preferences for the different graphical presentation styles. For those receiving non-colored charts, almost half of the patients did not have a preference (*χ*^2^ = 30.5, *p* < 0.001). For patients who received colored charts, bar charts were favored (*χ*^2^ = 49.2, *p* < 0.001).

**Table 2:** Preferences for graphical presentation styles

Non-colored charts	Patients^a^ (*N* = 232)	Medical specialists (*N* = 86)	Nurses (*N* = 141)^b^
	Frequency (%)	Frequency (%)	Frequency (%)
Bar charts	30.3	18.9	32.0
Line charts	19.9	21.6	18.0
No preference	49.8	NA	NA

Colored charts	Patients^a^ (*N* = 316)	Medical specialists (*N* = 86)	Nurses (*N* = 141)^b^
	Frequency (%)	Frequency (%)	Frequency (%)
Bar charts	38.9	12.2	4.0
Line charts	11.5	1.4	14.0
Heat map	19.8	45.9	32.0
No preference	29.9	NA	NA

^a^Numbers in this column add up to 200 % because patients either judged the non-colored or the colored charts. For both types of charts, the total adds up to 100 %

^b^Nurses and other health professionals

The mean number of correct answers to the twelve questions assessing objective understanding was 7.0 (59 %); for absolute scores, this was 2.0 (out of 4), for overall change 2.5 (out of 4), and for specific change 2.6 (out of 4). Functioning scales were better understood than symptom scales (absolute scores *t* = 3.08, *p* = 0.002; overall change *t* = 4.91, *p* < 0.001; specific change *t* = 9.93, *p* < 0.001). Table 3 shows, for each question, the percentage of participants with a correct response. These results did not differ significantly between the different graphical presentation styles (not shown in tabular form).

**Table 3:** Objective understanding (percentage of patients answering correctly)

Domain	Absolute score^a^	Overall change	Specific change
	(*N* = 316) (%)	(*N* = 548) (%)	(*N* = 548) (%)
Physical functioning	44.4	62.1	72.2
Emotional functioning	60.4	72.8	76.7
Fatigue	42.8	54.6	52.6
Pain	51.9	60.5	57.3
Total^b^	49.8	63.5	65.3

^a^Calculated for group C/D/E (colored charts) only

^b^These numbers reflect the percentage of correct answers (mean number of correct answers divided by the maximum possible number of correct answers)

Objective understanding did not differ significantly between men and women. Younger patients (below the median age of 62 years) were better in understanding specific change than older patients (*t* = 4.15, *p* < 0.001). For both specific change (*F* = 14.705, *p* < 0.001) and overall change scores (*F* = 6.591, *p* = 0.002), we found an effect of educational level. Post hoc pairwise comparisons indicated that patients with a university or college degree were better in understanding specific and overall change scores than patients with lower educational levels.

A much higher percentage (83 %) of respondents reported that they found the graphs (very) easy to understand; this did not differ between groups (*χ*^2^ = 6.76, *p* = 0.149).

With regard to directional indicators, one-third of patients (34.8 %) did not have a preference and 34.4 % preferred the green and red arrows. These percentages were significantly higher than the percentage of patients preferring the green arrow indicating better scores (23.4 %) and plus and minus signs (7.3 %) (*χ*^2^ = 102.1, *p* < 0.001).

#### General aspects of PRO data collection and use

The majority of patients (75.8 %) believed that PROs are a good way to provide their professional caregivers with information about how they are feeling. Completing such a questionnaire at home was favored over completing it in the hospital (39.0 vs. 16.2 %; 44.8 % had no preference). About half of the patients (46.9 %) were willing to spend 15–30 min to complete PROs and would prefer to receive oral feedback on PRO results from a health professional (49.7 %). The largest group of patients would like to complete PROs once a month during treatment (35.9 %) and once every 3 months after treatment (30.0 %). The preferred comparison group was one’s own previous results (40.9 %). Further details are given in Table 4.

**Table 4:** General aspects of PRO data collection and use

	Patients (*N* = 548) (%)	Health professionals (*N* = 227) (%)
Frequency during treatment		
Never	6.3	5.1
Every week	16.9	7.0
Every 2 weeks	18.0	12.7
Every month	35.9	47.1
Less than once a month	20.9	28.0
Frequency after treatment		
Never	9.7	5.0
Every month	20.5	4.4
Every 3 months	30.0	47.8
Every 6 months	23.2	31.4
Every year	16.5	11.3
Preferred comparison group^a^		
None	41.3	6.6
Other cancer patients	33.7	26.9
Healthy individuals	15.5	24.7
Previous personal results	40.9	55.5

^a^Percentages may exceed 100 % because giving more than one answer was allowed

### Health professional survey

#### Sociodemographics

A total of 227 health professionals completed the online questionnaire. Their mean age was 45.2 (SD 10.8) years, and 76.4 % was female. The largest group consisted of nurses (53.7 %) followed by medical specialists (37.9 %; e.g., medical oncologists, radiotherapists, surgeons, pulmonologists) and paramedical professionals (8.4 %; e.g., physical therapists, social workers). For analysis purposes, we combined nurses, nurse specialists, and paramedical professionals into a single group (“nurses and other health professionals”) and compared this combined group with medical specialists.

#### Graphical presentation styles

Thirty-two percentage of all health professionals indicated that they had previously used PROs (typically QoL questionnaires) in a clinical study, and 48.0 % reported using PRO results to inform patients about the possible (adverse) effects of treatment. About half of the professionals (56.3 %) had used individual-level QoL information in daily clinical practice.

Preferences for graphical display of QLQ-C30 results (all professionals) are shown in Table 2. There was a significant difference between professional groups. Forty-six percent of medical specialists preferred the heat map, whereas other health professionals preferred the heat map and non-colored bar charts equally (32 % for both) (*χ*^2^ = 16.9, *p* = 0.002).

The mean number of correct answers to the six questions assessing objective understanding was 4.7 (78 %) for medical specialists and 4.4 (74 %) for nurses and other health professionals. Understanding of overall change did not differ significantly between functioning and symptom scales (*t* = 1.68, *p* = 0.095). Specific change was understood better for functioning scales (*t* = 4.11, *p* < 0.001), whereas absolute scores were better understood for symptom scales (*t* = −3.92, *p* < 0.001). Table 5 shows the percentage of professionals who accurately interpreted the information summarized in the graphical displays. Understanding of overall and specific change scores did not differ significantly as a function of profession, but a significantly greater percentage of medical specialists than nurses and other professionals interpreted absolute scores accurately (72.6 vs. 52.9 %; *U* = 816.5, *p* = 0.024). Understanding did not differ significantly between those with and without previous experience with PROs. Regarding graphical presentation style, we found a significant difference for overall change scores, with the non-colored bar charts being interpreted correctly more often than the other graphical displays (*χ*^2^ = 16.9, *p* = 0.023; data not presented in tabular form).

**Table 5:** Objective understanding (percentage of professionals answering correctly)

Domain	Absolute score		Overall change		Specific change	
	Medical specialists	Nurses^a^	Medical specialists	Nurses^a^	Medical specialists	Nurses^a^
	(*N* = 86) (%)	(*N* = 141) (%)	(*N* = 86) (%)	(*N* = 141) (%)	(*N* = 86) (%)	(*N* = 141) (%)
Physical functioning	64.3	40.4	75.7	84.2	98.6	98.0
Fatigue	81.0	65.4	64.2	81.1	89.7	83.5
Total^b^	72.6	52.9	69.4	82.1	94.1	90.7

^a^Nurses and other health professionals

^b^These numbers reflect the percentage of correct answers (mean number of correct answers divided by the maximum possible number of correct answers)

A high percentage (85 %) of the health professionals indicated that the graphs were (very) easy to understand; this did not differ significantly between professions or graphical presentation styles. Both medical specialists (44.9 %) and other health professionals (64.4 %) preferred the green and red arrows as directional indicators. However, the second choice for medical specialists was the green “better” arrow (34.8 %), whereas for nurses, the second choice was the graph with the plus and minus signs (19.8 %) (*χ*^2^ = 24.47, *p* < 0.001).

About 13 % of health professionals responded to the open-ended question with a comment about the way in which the QLQ-C30 is scored, with higher scores being better for functional scales and worse for symptom scales. Some respondents indicated that they would prefer the use of a uniform direction for scoring, while others stressed the importance of highlighting this distinction more clearly to avoid confusion.

#### General aspects of PRO data collection and use

A large majority (87.8 %) of health professionals believed that PROs are a useful way to obtain information about how their patients are feeling. Nearly all professionals (96.3 %) indicated that it would be useful to access the results of PROs via the electronic medical record, and many (76.5 %) would wish to receive an alert when scores indicated a clinically relevant deterioration in functioning or increase in symptoms. Those who did not consider PROs to be useful reported having had negative experiences with such data, not having the time to review and discuss PRO results with their patients, and/or not wanting to bother their patients with completing questionnaires. Almost half of the professionals would like their patients to complete PROs once a month during treatment and once every 3 months after treatment. The majority preferred to compare a patient’s current scores with his/her previous scores. Further information is provided in Table 4.

## Discussion

In this study, we investigated cancer patients’ and health professionals’ understanding of and preferences for graphical presentation styles for individual-level PRO data obtained using the EORTC QLQ-C30 questionnaire. Patients’ objective and self-rated understanding were similar for the five graphical presentation styles, although they had a slight preference for bar graphs. Health professionals preferred heat maps, followed by non-colored bar charts and non-colored line charts. Their understanding of overall change was better for non-colored bar charts, and medical specialists were more accurate than other professionals in interpreting absolute scores. Self-rated understanding was substantially higher and did not differ significantly between professions or graphical presentation styles.

Compared with previous studies, the objective understanding of the patients in our study was relatively low; it varied from 42.8 to 76.7 %. In previous studies using group-level data, these figures did not fall below 80 % [8, 17]. As educational levels of patients appear to be comparable across the different studies, this is not likely to explain these differences. However, in a study using individual-level data, the percentage of correct answers varied from 64 to 96 % [18], which is also higher than the percentages we found. This rules out that the differences in observed understanding are caused by the use of group-level versus individual-level data. Possibly, the lower levels of understanding are due to the different types of graphical formats that were used in the studies.

Professionals’ understanding varied from 52.9 to 94.1 %, which is relatively low compared to the results of a recently published mixed-methods study, showing that oncologists answered 90–100 % of questions correctly [18]. This difference might be due to the fact that we included professionals with different backgrounds, whereas in the mixed-methods study, only oncologists were included. However, with some exceptions, professionals’ understanding of the PRO results presented graphically was much higher than that of patients’. We suspect that this may be due to their familiarity with interpreting data, in general, as well as to the fact that some of the health professionals had had previous experience with PROs, in general, and the QLQ-C30, in particular. Within the group of health professionals, we found that medical specialists were better in interpreting absolute scores than nurses and other health professionals, possibly because medical specialists are more accustomed to interpreting numerical data and charts. Many participating professionals indeed indicated that they had previous experience with PROs, for example in clinical practice. As we only recruited professionals from the Netherlands Cancer Institute, a comprehensive cancer center, these results may not be representative of health professionals, in general.

It is noteworthy that the self-rated understanding of both patients and health professionals was much higher than objectively measured understanding. Respondents may have answered the question assessing their self-rated understanding in a socially desirable way, providing an overly optimistic view. This is in line with two studies on lay understanding of medical terms [19, 20]. Self-rated understanding in this study did not differ as a function of graphical presentation style, whereas previous research has shown that line graphs were self-rated as easiest to understand [8].

Our findings regarding preferences are not in line with findings from studies on group-level data, which report that line graphs are preferred by patients and professionals [8, 10] or with a study on individual-level data in which line graphs were also preferred [18]. However, in those studies the selection was not made from a set of chart types fully comparable to the options used in our study. This discrepancy may reflect a methods effect; if different combinations of graphs would be used, preferences might also differ.

We found that both patients and professionals preferred PROs to be completed once a month during treatment and every 3 months after treatment. The higher frequency during treatment seems reasonable, given that one could expect more fluctuation and change in symptoms and functional health during this period. These findings are in line with the considerations of Snyder and colleagues regarding the implementation of PROs in clinical practice [11]. In addition, respondents in both groups indicated that they would prefer to compare current scores with a patient’s previous scores. Detecting worsening of symptoms and deterioration in functioning is particularly important in order to provide relevant care in a timely manner.

Our study has several limitations that need to be considered. First, although we investigated five graphical presentation styles, these did not represent all possible styles. Furthermore, patients were not shown all types, but only the non-colored or the colored ones (to prevent an exposure effect). Second, we only used hypothetical data, which might have led to an underestimation of objective understanding. Some patients explicitly indicated that the graphs were not representative of their health situation at the indicated time points. This suggests that these patients may have answered the questions with their own health status in mind, which could have been different from the health status shown in the graphs. Possibly their interpretation would be more accurate if these patients were to be provided with graphs reflecting their own health status. Another limitation of the study is that we were only able to survey health professionals from a single hospital.

Our study also had a number of strengths, including the use of a variety of graphical presentation styles, the use of colored and non-colored graphics, and inclusion of patients from a number of countries, with different diagnoses, and both on- and off-treatment. We were also able to include a sizeable number of health professionals representing a variety of professions.

Because particularly patients’ objective understanding was relatively low, it is important to learn more about how patients interpret and understand their individual graphically displayed PRO results. What are they thinking when they view such results? What information draws their attention? What do they understand and what do they not understand? These questions could be addressed via interviews in which patients are asked to verbalize what they are thinking when presented with graphs to interpret (a “think aloud” exercise [21]) and/or to reflect on their thinking process in retrospect). The results of such a qualitative study could be used to develop educational materials to help patients better understand their PRO results. For example, a tutorial video could be developed in which instructions are provided about the interpretation of PRO results. Special attention should be paid to the interpretation of functioning versus symptom scales, as our study as well as another study [18] showed differences in understanding between these types of scales. Such a video could also include a test to assess whether a patient fully understands the graphs. Comparable materials could be developed for professionals. Such a tutorial should focus not only on interpretation, but also on how to best provide care to and/or refer patients with clinically relevant QoL scores. In a previous study, professionals indeed indicated that they required help interpreting QoL data, and especially the clinical relevance of those data [10].

## Conclusion

In this study, we investigated patients’ and health professionals’ understanding of and preferences for different graphical presentation styles of individual PRO results. Our results indicate that although patients and health professionals generally believe that they understand PRO results summarized in graphical displays, in fact, their level of understanding is considerably lower. Thus, future studies are needed to better understand the causes of misunderstanding and to determine how to optimally present PRO results in a graphical form. This information could be used to develop educational materials that help to optimize interpretation. Because we did not find a clear preference for a certain graphical presentation style, choosing different styles for different individuals should be considered. This could be facilitated by using electronic systems to collect and feedback PROs.
